# Resonant Frequency Response to Mechanical Loading in Conformal Load-Bearing Antenna Systems

**DOI:** 10.3390/s25051323

**Published:** 2025-02-21

**Authors:** Shouxun Lu, Kelvin J. Nicholson, Joel Patniotis, John Wang, Wing Kong Chiu

**Affiliations:** 1 Department of Mechanical & Aerospace Engineering, Monash University, Clayton, VIC 3800, Australia; wing.kong.chiu@monash.edu; 2 Platforms Division, Defence Science and Technology Group, Fishermans Bend, VIC 3207, Australia; kelvin.nicholson@defence.gov.au (K.J.N.); joel.patniotis@defence.gov.au (J.P.); john.wang@defence.gov.au (J.W.)

**Keywords:** conformal load-bearing antenna system (CLAS), resonance frequency, relative permittivity, glass fibre-reinforced polymer (GFRP), fatigue response, mechanical testing

## Abstract

This study investigates the impact of mechanical loading on the electromagnetic performance of conformal load-bearing antenna structures (CLASs), focusing on the resonant frequency response. Using 6-ply [0/90] GFRP as the CLAS substrate, the research evaluated the effects of two mechanical loading scenarios: the quasi-static uniaxial tensile test and cyclic fatigue. The quasi-static tests explore the response of CLASs to significant elongation, while the cyclic fatigue tests simulate localised damage propagation under operational loads. The results from the quasi-static tests demonstrated that the dominant effect under uniaxial tensile loading is the increase in substrate permittivity due to damage, causing a decrease in resonant frequency. The cyclic fatigue tests employed two configurations: removeable antenna patch (RAP), which isolates the antenna from mechanical loading to focus on substrate damage; and surface-mounted antenna patch (SMAP), which examines the combined effects of substrate damage and antenna elongation. The RAP results showed a consistent correlation between substrate damage and resonant frequency decrease, while SMAP demonstrated complex frequency behaviour due to competing effects of substrate damage and antenna elongation. A comparison with [±45]_6_ GFRP results showed that the resonant behaviour remained consistent regardless of ply configuration during the initial damage accumulation induced by cyclic fatigue. However, with significant elongation in quasi-static tests, resonant frequency behaviour was affected by the specimen’s ply configuration, with substrate permittivity changes due to mechanical loading being the dominant factor. These findings provide valuable insights into the relationship between damage sustained by the CLAS system and resonant frequency shifts, providing critical information for predicting CLAS’s reliability and service life.

## 1. Introduction

**Figure 1:**
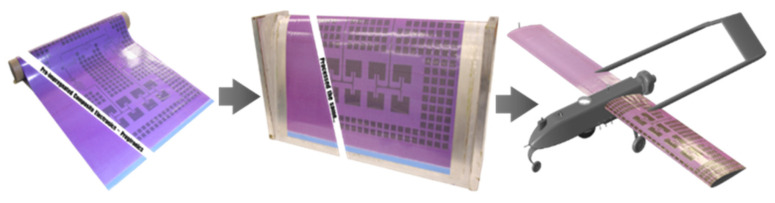
CLAS concept. Prepreg composite material with embedded electromagnetic traces can be cured in aerospace composite structures [2].

With the rapid advancement of UAVs in the aerospace industry, there is a significant need for increased functionality and efficiency despite volume constraints [1], necessitating greater integration and miniaturisation of electronics in UAV systems. Multifunctional composite structures (MFCSs) are essential research fields with large-scale applications in the aerospace, automotive, and sports sectors. Conformal load-bearing smart skin (CLSS), a subset of MFCSs, has attracted interest recently. With the growing demand for the integration and miniaturisation of UAV systems, CLSSs offer a promising solution. Specifically, conformal load-bearing antenna structures (CLASs), a recent development within CLSSs, focus on integrating radiofrequency devices into the composite skins of airframes. (see Figure 1).

Traditional communication and radar elements on aerostructures are considered separate sub-systems and have independent design and manufacturing considerations. It reduces synergy when optimising electromagnetic and structural requirements. Additionally, conventional radiofrequency devices are externally mounted, protruding into the airflow, disrupting aerodynamics, increasing drag, and reducing environmental tolerance during flight. In contrast, the CLAS system embeds these devices directly into the skin of load-bearing structures with minimal mechanical performance degradation. This integration reduces drag, which potentially improves fuel efficiency, enhances structural integrity, and reduces the radar cross-section (RCS) of the UAV, thereby enhancing its stealth capabilities. Strategically positioning radiofrequency elements along the exterior can further improve antenna performance. Additionally, the system is significantly more resilient to environmental damage than conventional antenna structures [3], leading to lower maintenance costs and increased operational reliability.

Material selection is a crucial aspect of designing and fabricating CLSSs, driven by the need to fulfil additional functional requirements. The chosen material must exhibit flexibility and strength comparable with the rest of the structure to ensure seamless integration. In CLASs, the substrate plays an important role in determining the performance of embedded communication elements.

Traditionally, CLAS designs feature a carbon fibre composite structural skin and an electrically insulating, radar-transparent window, often fabricated from glass fibre-reinforced polymer (GFRP) [4]. GFRP offers mechanical properties comparable with carbon fibre composites [5] and is used in fabricating UAVs and aerospace structures. Moreover, as reported in [6,7], GFRP’s radar transparency and dielectric stability ensure that its use as an antenna substrate does not adversely affect antenna efficiency, making it a highly suitable material for integrating antenna elements while maintaining CLAS’s structural and electromagnetic performance.

**Figure 2:**
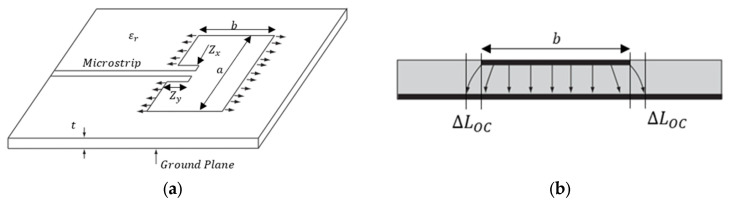
Schematic of: (**a**) geometry of a conventional microstrip antenna patch on a substrate with a thickness of t; (**b**) the fringing field, which extends beyond antenna edges [2].

In addition, to enhance the utility of CLASs, a microstrip antenna, widely regarded as one of the best options for modern communication systems, is selected for CLAS applications. This choice is driven by its numerous advantages, including ease of manufacture, adaptability to both planar and non-planar surfaces, and high customizability to meet specific design requirements [1,7,8]. An example of a CLAS is the conventional microstrip antenna shown in Figure 2a.

Equation (1) shows the relationship between the resonant frequency, $f_{r}$, for a microstrip antenna, the total effective length $b+2∆L_{OC}$ of the CLAS, and the dielectric properties $\epsilon_{eff}$ of the substrate (GFRP). The schematic representation of total effective length $b+2∆L_{OC}$ is shown in Figure 2b. The fringing fields extend beyond the physical boundaries b of the antenna patch. This extension causes the total effective length of the cavity to be greater than the physical length of the antenna patch. Specifically, an additional effective length is added at each edge due to the microstrip open-end effect.

(1) $$f_{r}=\frac{c}{2\sqrt{\epsilon_{eff}}\left(b+2∆L_{OC}\right)}$$

where c is the speed of light in vacuum, b is the dimension of the CLAS (shown in Figure 2), $∆L_{OC}$ is the open circuit length extension defined in Equation (2), and $\epsilon_{eff}$ is the effective permittivity of the substrate defined in Equation (3) [9].

(2) $$\frac{∆L_{OC}}{t}=0.412\frac{\left(\epsilon_{eff}+0.3\right)\left(\frac{a}{t}+0.264\right)}{\left(\epsilon_{eff}-0.258\right)\left(\frac{a}{t}+0.813\right)}$$

where a and t are the physical properties of the CLAS (shown in Figure 2).

(3) $$\epsilon_{eff}=\frac{\epsilon_{r}+1}{2}+\frac{\epsilon_{r}-1}{2}{\left(1+\frac{10t}{a}\right)}^{-0.5}$$

where $\epsilon_{r}$ is the relative permittivity, which is defined as the ratio of the material’s permittivity (ϵ) to the permittivity of free space $\epsilon_{0}$.

**Figure 3:**
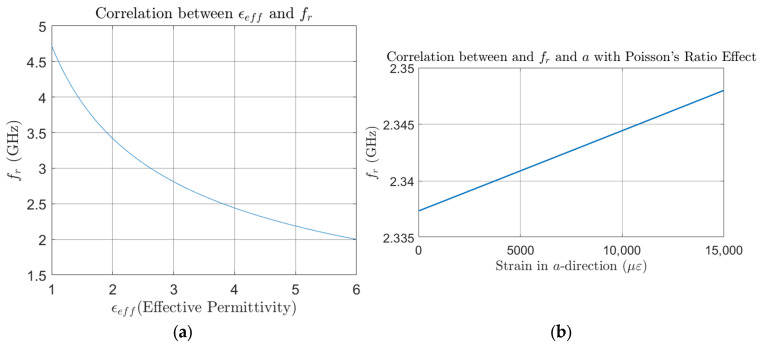
Correlation between: (**a**) effective permittivity of the substrate $\epsilon_{eff}$ and resonant frequency $f_{r}$; (**b**) strain in dimension a and resonant frequency $f_{r}$, considering the Poisson’s Ratio.

The relationship between effective permittivity $\epsilon_{eff}$, strain in dimension a, and the resonant frequency $f_{r}$ of the CLAS system, based on the properties of the system employed in this study, is shown in Figure 3a,b. These figures show a clear inverse correlation, where an increase in $\epsilon_{eff}$ leads to a corresponding decrease in $f_{r}$. This relationship highlights how the permittivity change influences the resonant frequency of the CLAS system.

### Potential Parameters That Affect the CLAS Resonant Performance

**Figure 4:**
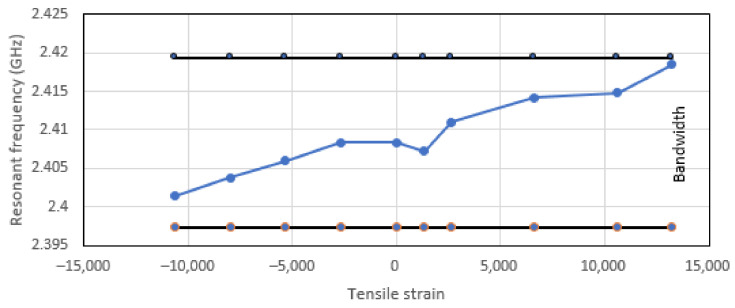
Resonant frequency response to uniaxial tensile and compressive strain along the fibre’s orientation [2].

The antenna’s physical geometry and the substrate’s electromagnetic properties will define the CLAS’s resonant frequency. According to Equation (1) mentioned above, changes in the geometry due to mechanical loading can alter the resonant behaviour. This relationship was explored in research by Healey et al. [2], which investigated the resonant frequency response of CLASs under various mechanical loading conditions, such as uniaxial, biaxial, and twisting loads, through numerical simulations. The study only considered the effect of mechanical loading on the antenna geometry and substrate thickness. The results highlighted that with uniaxial tension being applied along dimension a, which affects dimension b, due to the Poisson’s ratio, the resonance frequency of CLAS shows a clear increasing trend as the tensile strain applied to the GFRP substrate progresses, as shown in Figure 4. This behaviour is consistent with Equation (1) above, which states that the changes in the patch’s physical dimensions directly impact the resonance, leading to frequency shifts. This result also reveals that the resonant frequency still falls within acceptable bandwidth after the tensile strain reaches 15,000 µε.

**Figure 5:**
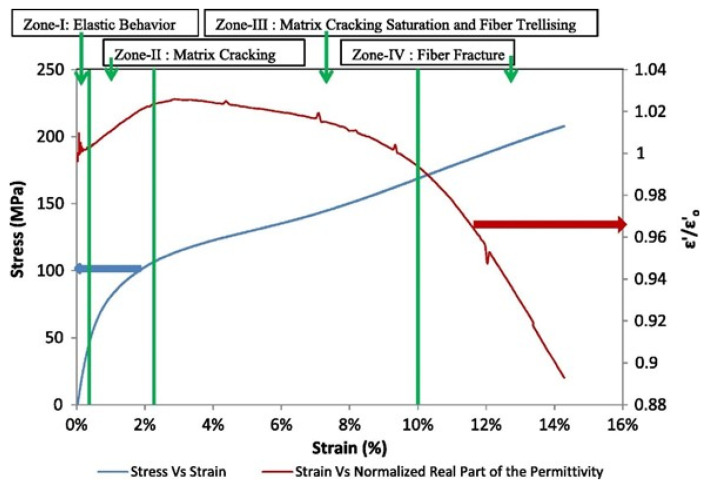
Response of the dielectric property and stress to strain under the uniaxial tensile test [10].

In addition, when applied to aerostructures, the mechanical loading will influence the CLAS’s geometry and damage the CLAS’s substrate. The relationship between damage sustained by the substrate and its dielectric properties has been a research focus in recent publications. For instance, Raihan R. et al. [10] conducted an in-depth study on the dielectric behaviour of [±45]_5_ GFRP under the quasi-static tensile test. The results (see Figure 5) illustrated that the effective permittivity $\epsilon_{eff}$ increased during the initial stage of damage accumulation, which is attributed to microcracking in the matrix. As the microcracking reached saturation, the permittivity began to decline, with a significant reduction observed when fibre fracture occurred. Similar results were reported in computational publications [11,12,13] and experimental data in [14]. Therefore, based on Equation (1), when CLAS underwent a quasi-static test, the resonant frequency of the CLAS initially decreased, followed by an increase until specimen failure. The work reported by Lu et al. [15] confirmed this trend. In this work, they examined the resonant behaviour of the CLAS with [±45]_6_ GFRP as the substrate under both quasi-static tension and cyclic fatigue tests. This investigation also introduced a novel method involving a removable antenna patch that isolates the patch from mechanical loading, thereby highlighting the primary influence of substrate damage on the resonant behaviour of the CLAS. The results from the quasi-static test demonstrated a similar trend in resonant frequency shifts consistent with predictions based on fundamental resonant frequency equations and the literature on permittivity. Additionally, the results from the cyclic fatigue tests indicated that the resonant frequency of the CLAS gradually shifted downward as substrate damage accumulated, evidenced by a progressively expanding whitened region near the fixed support as fatigue cycles increased.

The literature confirms that mechanical loading significantly impacts the dielectric properties of ±45° GFRP which, in turn, influences the electromagnetic performance of the CLAS. GFRP is highly anisotropic, meaning its material properties vary depending on the fibre alignment. Different fibre orientations can alter strain distribution, directly affecting damage accumulation and the electromagnetic response under mechanical loading. Therefore, understanding how fibre orientation influences both the structural integrity and electromagnetic properties of the CLAS under various loading conditions is essential for optimising material selection and antenna placement.

To address this, the present study incorporates the existing literature on the electromagnetic behaviours of GFRP with different fibre orientations under mechanical loading.

**Figure 6:**
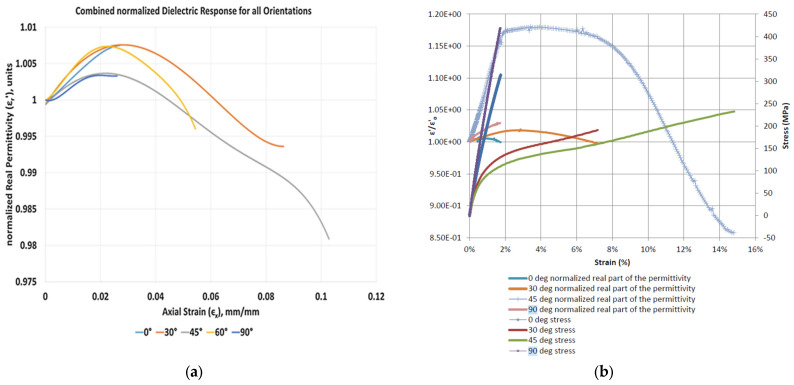
Dielectric response curves for: (**a**) [(0°)]_8_ GFRP specimens cut at five orientations [14]; (**b**) woven [0/90°]_5_ GFRP specimens cut at four orientations [16].

Vamsee. V. et al. utilised a cured GFRP panel of [(0°)]_8_, cut into five different orientations. These specimens underwent uniaxial tensile testing, with electrode blocks being attached to the gage region for in situ dielectric measurements [14]. The results, shown in Figure 6a, revealed that fibre orientations of 30°, 45°, and 60° exhibited an initial increase in permittivity, followed by a decrease until failure. In contrast, 0° and 90° fibre orientations displayed a relatively monotonic positive correlation between strain and permittivity. Similar results were also reported in the study conducted by Raihan, M.R. [16], in which uniaxial tensile tests were conducted on woven [0/90°]_5_ GFRP specimens. The result in Figure 6b demonstrated a generally positive trend in permittivity with increasing strain under tensile loading at principal angles of 0° and 90°, respectively. According to Equations (1) and (2) and Figure 3a, the increase in permittivity leads to a decrease in the CLAS’s resonant frequency under the condition that only GFRP is subjected to the loads.

These above findings demonstrate that mechanical loading can influence the CLAS’s performance through two primary mechanisms: (1) geometric deformation of the antenna patch; and (2) changes in the dielectric properties of the GFRP substrate due to material degradation. However, previous research has predominantly focused on isolated aspects of the system rather than addressing the combined effects of mechanical loading on the overall performance of the CLAS. A holistic understanding of how antenna deformation and substrate material degradation interact under mechanical stress remains lacking.

Furthermore, the electromagnetic response of the CLAS to mechanical loading varies with fibre orientation. Studies show that [±45°] GFRP undergoes significant changes in permittivity, whereas [0/90°] GFRP exhibits a more gradual increase in the dielectric response under tensile loads. This variation suggests that fibre orientation plays a crucial role in the electromagnetic stability of the CLAS and must be carefully considered in its design and application.

However, the combined effects of mechanical loading, material degradation, and fibre orientation on CLAS performance remain unexplored, posing a critical research gap that requires further investigation. To ensure the successful integration of the CLAS into aerostructures, it is essential to study how mechanical deformation, dielectric property variations, and fibre orientation collectively impact the CLAS’s structural and electromagnetic behaviour. Addressing this gap is crucial for realising the full potential of CLASs and their reliability for long-term aerospace applications.

The motivation for this study stems from the need to test and validate CLAS performance under various mechanical loading conditions, including uniaxial tension and localised cyclic fatigue. This study aims to bridge the gap between structural mechanics and electromagnetic performance in CLASs by analysing both substrate-only effects and combined antenna–substrate system responses. In addition, this paper investigates a CLAS integrated with [0/90°] GFRP, providing further insights into its mechanical and electromagnetic behaviours. The findings will contribute to optimising antenna placement, structural resilience, and electromagnetic reliability in future aerospace designs.

This research is essential for ensuring that CLAS-integrated UAV airframes maintain consistent and reliable communication performance despite mechanical deformations encountered during flight operations. A comprehensive understanding of these interactions will be crucial for designing robust, load-bearing antenna structures that retain electromagnetic performance under real-world operational conditions.

## 2. Methodology

### 2.1. Specimens

**Figure 7:**
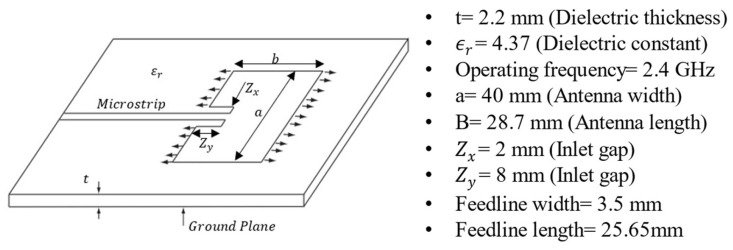
Detailed dimensions of the microstrip antenna patch.

This research utilised a microstrip antenna patch as the radiofrequency element for the CLAS. The antenna patch in this study shares the same design as in [15], intended to resonate at 2.4 GHz with an acceptable bandwidth of 22 MHz to facilitate the IEEE 802.11 local area network standard protocol. Figure 7 shows the detailed dimensions of the antenna. Non-woven fibre mats were selected as the antenna material due to their high porosity, allowing effortless consolidation with traditional composite structures. This copper-coated veil was produced through in-house electroplating of a nickel veil by the Australian Defence Science and Technology Group (DST Group). The material, with a nominal thickness of 0.15 mm, was then laser-cut to replicate the design dimensions.

The CLAS system’s substrate was a series of six plies of plain-weave GMS EP-280 S-Glass glass fibre-reinforced polymer (GMS composites, Chadstone, VIC Australia), oriented at 0° and 90°. A similar microstrip patch antenna and GFRP combination were reported in [15,17].

This paper introduced three types of CLAS specimens to investigate the response of CLAS to damage systematically. All specimens were stored in a temperature- and moisture-controlled environment to minimise the potential impact of environmental temperature and humidity. Based on the different requirements for each specimen, the preparation methods for each configuration type were slightly different, as elaborated below.

#### 2.1.1. Configuration A: Integrated Antenna Patch (IAP)

**Figure 8:**
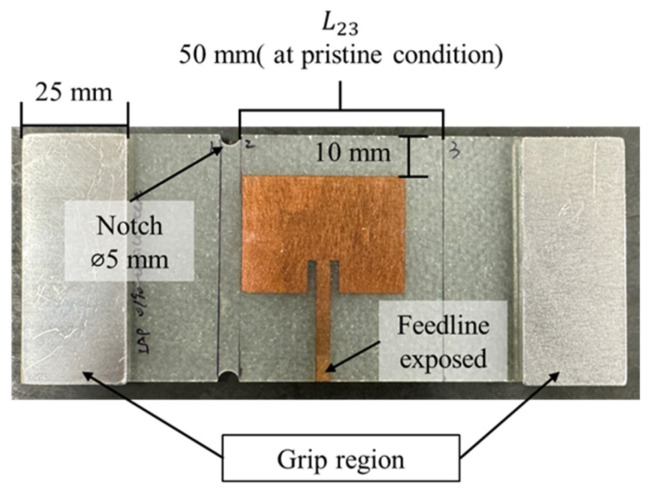
Configuration A: integrated antenna patch (IAP) with notches and aluminium tabs on each end for tensile tests.

In this configuration, the antenna patches were placed onto the laminate layers before the curing process, mirroring the preparation of the CLAS in real-world applications. After curing, the test plate was cut into multiple specimens, each measuring 146 mm by 60 mm, with the antenna being located at the centre using CNC machining. The feedline of the antenna was exposed through laser ablation. Two pairs of aluminium tabs were bonded to the ends of each specimen, with rough material at the interfaces to provide sufficient friction and gripping area for subsequent tests (Figure 8). The bonding between the antenna patch and the substrate ensures that both components simultaneously experience the strain induced by mechanical loading.

#### 2.1.2. Configuration B: Removeable Antenna Patch (RAP)

**Figure 9:**
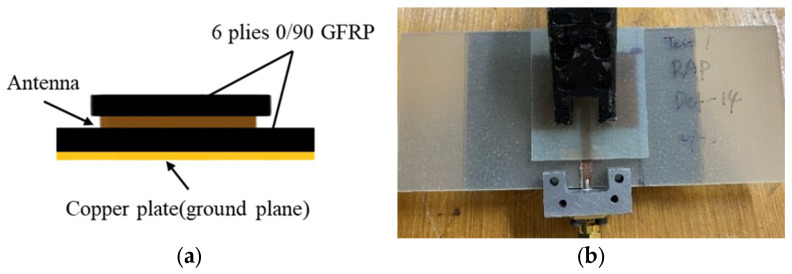
Configuration B: removeable antenna patch (RAP): (**a**) schematic diagram; (**b**) experimental setup with SMA connect for the S11 measurement.

The schematic design of Configuration B is shown in Figure 9a. An antenna patch was bonded to a separate small 6-ply GFRP panel. This antenna assembly was then applied to a cured [0/90]_6_ GFRP cut into the same dimension as Configuration A: IAP, using a clamp, as shown in Figure 9b. The removable antenna assembly was removed before it underwent mechanical loading. This experimental method preserves the pristine condition of the antenna patch, ensuring that only the substrate incurs damage from the mechanical loading. By isolating the antenna from mechanical effects, we can effectively analyse the impact of substrate damage on the resonant frequency of the CLAS.

#### 2.1.3. Configuration C: Surface-Mounted Antenna Patch (SMAP)

**Figure 10:**
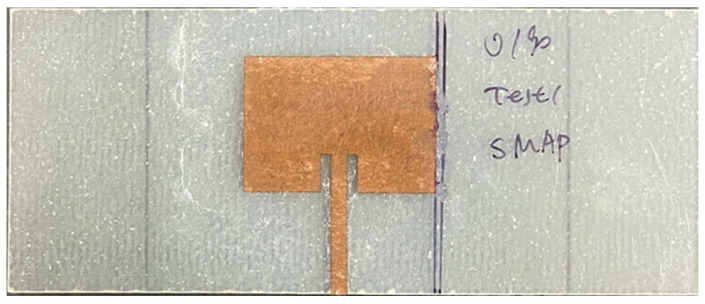
Configuration C: surface-mounted antenna patch (SMAP).

Configuration C (SMAP), as shown in Figure 10, was introduced to supplement the study and to compare the results from the integrated antenna patch designated as “IAP” above. In this SMAP configuration, cyanoacrylate bonded antenna patches to the substrate’s centre.

### 2.2. S11 Measurement and Comparison Between Configurations

**Figure 11:**
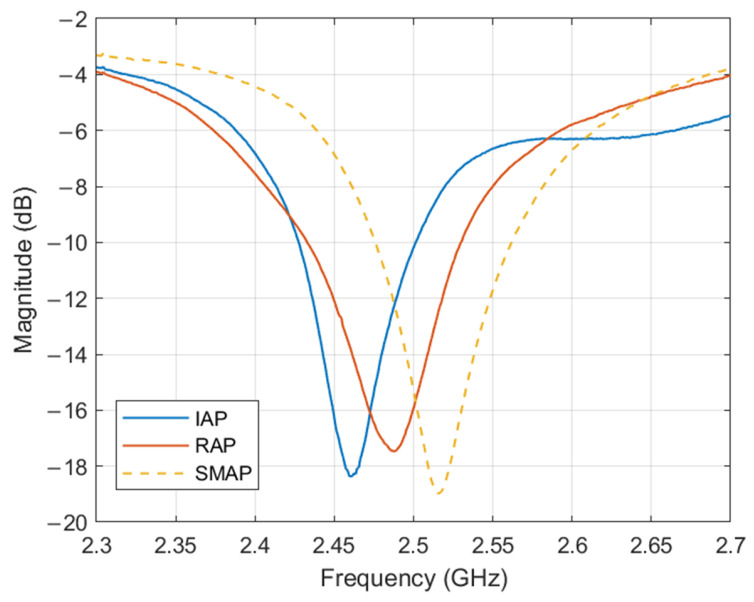
S11 curve for IAP, RAP, and SMAP.

Due to the mechanical loadings applied in this research, the specimens are expected to sustain damage. Consequently, the conventional ground plane (copper foil) used in antenna systems is unsuitable because it is prone to cracking and debonding from the substrate under mechanical stress [18,19], leading to significant degradation in electromagnetic performance. A thin copper plate was utilised as the ground plane and applied to the back of the substrate while measuring the antenna input reflection coefficient, S11, to maintain a consistent ground plane. The measurements were conducted using a NanoVNA V2 Plus 4 with an SMA connector attached to the antenna feedline, as shown in Figure 9b. Figure 11 presents the S11 plots for all three configurations, with resonant peaks ranging between 2.45 GHz and 2.55 GHz. Despite slight frequency shifts between the conventional CLAS preparation (Configuration A: IAP) and the other configurations, the RAP and SMAP curves demonstrated good impedance matching, with a magnitude drop at the resonance frequency exceeding 10 dB, confirming that the results obtained from Configuration B (RAP) and Configuration C (SMAP) are reliable for subsequent experimental tests.

### 2.3. Experimental Setup

This paper is a follow-up research study for that reported in [15] that investigated the resonant response of CLAS with [±45]_6_ GFRP under the quasi-static tensile test and cyclic fatigue. This study extends similar mechanical testing to specimens with [0/90]_6_ GFRP. The detailed experimental setup was designed based on preliminary tensile test results, as described below. Whilst the applied loading on GFRP test specimen in the work in [15] will lead to damage sustained predominantly by the matrix material, in the work described in this paper, the glass fibre carries the applied loading.

**Figure 12:**
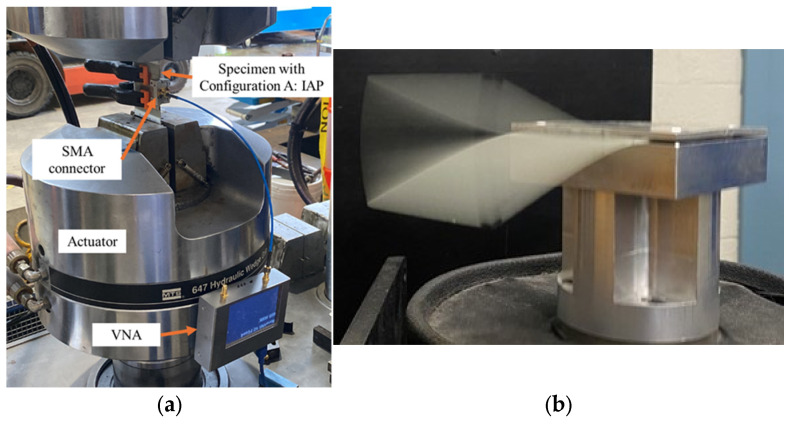
Experimental setup: (**a**) quasi-static tensile test with the VNA attached to an actuator; (**b**) first mode vibration of GFRP induced by a mechanical shaker.

Quasi-static tensile experiments were conducted on the three specimens with Configuration A: IAP. Two pairs of aluminium tabs, bonded with a rough interface material, were attached to the specimens to ensure sufficient gripping force. Notches with a 5 mm diameter were created 2.5 mm away from the antenna patch at the top and bottom edge of the specimens to localise the damage and avoid unexpected specimen failure at the grip region. The quasi-static tensile tests, with the setup illustrated in Figure 12a, were performed using MTS 810 machines at a displacement rate of 0.5 mm/min. At each pre-set displacement step, the length $L_{23}$ between lines 2 and 3 and the S11 parameters were measured while the specimen remained in the grips. Strain ε was calculated using Equation (4) to quantify the damage sustained around the antenna. This experimental procedure was repeated until specimen failure occurred.

(4) $$\varepsilon =\frac{∆L}{L_{23}}$$

where ε is the strain at the location of the antenna patch; $L_{23}$ is the original length between lines 2 and 3. ∆L is the change in $L_{23}$, measured after each step.

In addition to the research on resonant response to relatively rapidly developing damage and significant specimens’ elongation under quasi-static tensile testing, we conducted a series of cyclic fatigue tests to investigate how the S11 parameter changes as damage approaches the antenna patch. The damage was highly localised and identifiable through visual inspection of the specimen. The specimens were mounted on an electromechanical shaker during the cyclic fatigue tests, as shown in Figure 12b. They were excited at their first natural frequency mode, with a vibration amplitude that delivered a bending tensile strain of approximately 5000 µε at the fixed edge. S11 measurements were conducted every 400,000 cycles during the fatigue experiments. We tested three specimens each for Configuration B: RAP and Configuration C: SMAP.

## 3. Result

### 3.1. Preliminary Tensile Test

**Figure 13:**
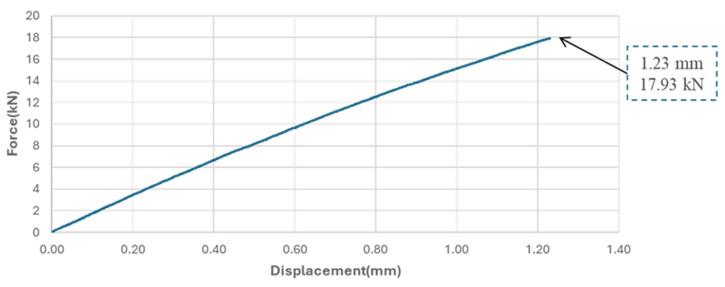
Force–Displacement curve for the plain-weave [0/90]_6_ GFRP specimen under tensile loading.

Figure 13 shows the displacement–load correlation obtained from a tensile to failure test on a 6-ply [0/90] plain-weave GFRP specimen. Because the loading direction is along its principal direction (0 degrees), the result demonstrates a close to linear behaviour with specimen failure at a force of 17.93 kN, accompanied by a displacement of 1.23 mm. The following load–displacement curve was used to set the displacement levels in subsequent quasi-static tensile tests.

### 3.2. Quasi-Static Tension Test

**Figure 14:**
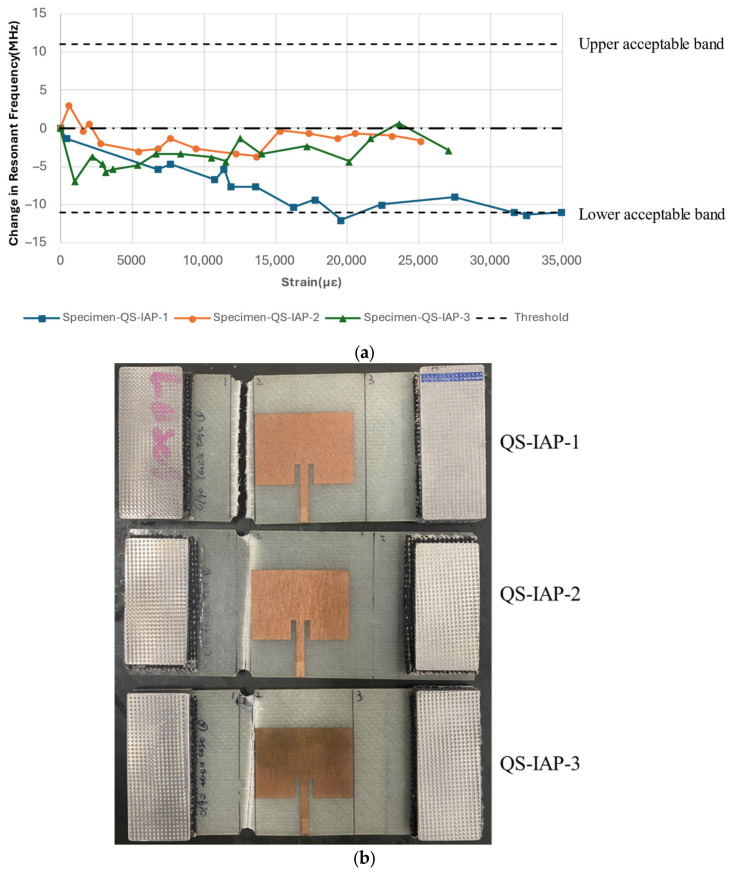
Specimen with Configuration A: IAP: (**a**) resonant frequency shift due to uniaxial tensile; (**b**) different failure modes between specimens.

The previous literature reports two distinct trends in the resonant frequency behaviour of CLASs under uniaxial loading in the fibre principal direction: (a) The changes in the geometry of the CLAS due to mechanical effects when subjected to a tensile load will lead to an increase in the resonant frequency; and (b) the material response of the GRFP due to large tensile load will give rise to an increase in permittivity and will lead to a decrease in resonant frequency. Our results, presented in Figure 14a, show the resonant frequency response to strain for the specimen configured as Configuration A: IAP.

For all three specimens, the resonant frequency demonstrated a general decreasing trend with a strain value reaching 11,000 με, which is unlikely to be attained in an aerospace structure. However, the resonant frequency of these specimens is still within the acceptable bandwidth after reaching this high strain level, proving the CLAS system’s resilience to mechanical loading.

In addition, as described by Equation (1), an increase in the GFRP’s permittivity is necessary to lower the resonant frequency of the CLAS. The observed decrease in the CLAS’s resonant frequency at these strain levels aligns with the increase in [0/90] GFRP permittivity shown in Figure 6b. Considering the opposing effects on the resonant frequency of the CLAS during the tensile test, which are frequency increases caused by the elongation of the antenna patch and frequency decreases resulting from the substrate’s permittivity response to damage, the overall resonant frequency exhibited a decreasing trend. This observation confirms that, under uniaxial loading along the substrate’s principal direction, the increase in permittivity due to damage is the dominant factor influencing the resonant frequency.

After 11,000 με, the resonant frequency measured from Specimen-QS-IAP-1 continued to decrease, reaching the lower acceptable limit before the specimen failed at approximately 35,000 με. The other two specimens with the same configuration exhibited a notable difference in the resonant frequency–strain correlation, displaying fluctuations between 0 and −5 MHz relative to the frequency measured under pristine conditions despite undergoing the same experimental setup. These specimens failed at strains of 25,150 με and 27,020 με, respectively. As shown in Figure 14b, the failure modes of these two specimens reveal that cracks propagated and occurred beneath the antenna patch, which is markedly different from the failure mode of QS-IAP-1, where the crack connected two notches in the vicinity of the antenna patch. This difference in crack propagation paths may be attributed to a slight misalignment between the loading and principal fibre directions. However, the ability of the CLAS to withstand mechanical loading and maintain performance is evident from the behaviour of the resonant frequency, which fluctuates within the acceptable bandwidth before the specimens’ failure.

### 3.3. Cyclic Fatigue Tests

The results presented above are consistent with the previous literature on how the permittivity of GFRP responds to uniaxial loading, which in turn affects the resonant performance of the CLAS. This finding also explains how uneven loading can alter the system’s resonant frequency. However, extreme specimen elongation to failure is unlikely under typical service conditions.

To simulate realistic scenarios, the subsequent research focuses on the behaviour of the CLAS in response to fatigue damage. This damage was induced using a mechanical shaker to create localised damage near the antenna patch through cyclic vibration. Two types of specimens were utilised. Configuration B: RAP was employed solely to investigate the effects of fatigue damage on the substrate. Configuration C: SMAP, in which the antenna patch is securely bonded to the substrate, was used to study the resonant frequency behaviour under mechanical damage affecting both the geometry of the specimen (antenna patch) and the dielectric properties of the substrate.

#### 3.3.1. Configuration B: Removeable Antenna Patch (RAP)

**Figure 15:**
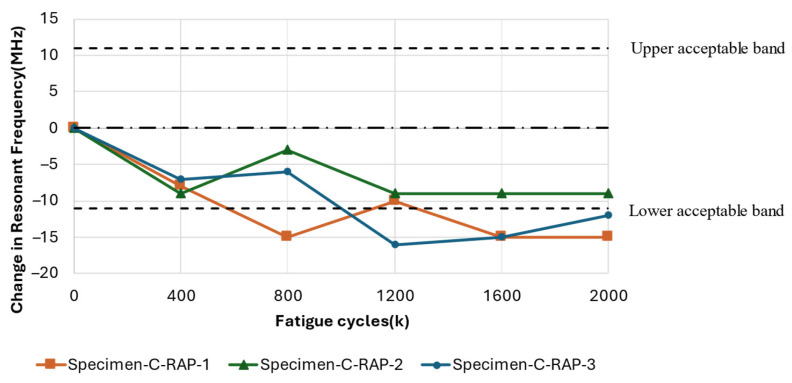
Resonant frequency shift of the CLAS due to substrate fatigue with Specimen Configuration B: RAP.

Figure 15 shows a general decreasing trend in resonant frequency with increasing fatigue cycles experienced by all three specimens. This trend aligns with previous findings that associate changes in dielectric properties with damage induced by mechanical loading of the substrate. Specifically, the resonant frequency of Specimen-C-RAP-2 decreased by 9 MHz from its pristine condition but remained within the acceptable bandwidth after the test. In contrast, Specimen-C-RAP-1 and Specimen-C-RAP-3 exhibited decreases of 15 MHz and 12 MHz, respectively, deviating outside the acceptable bandwidth after 2 million fatigue cycles.

**Figure 16:**
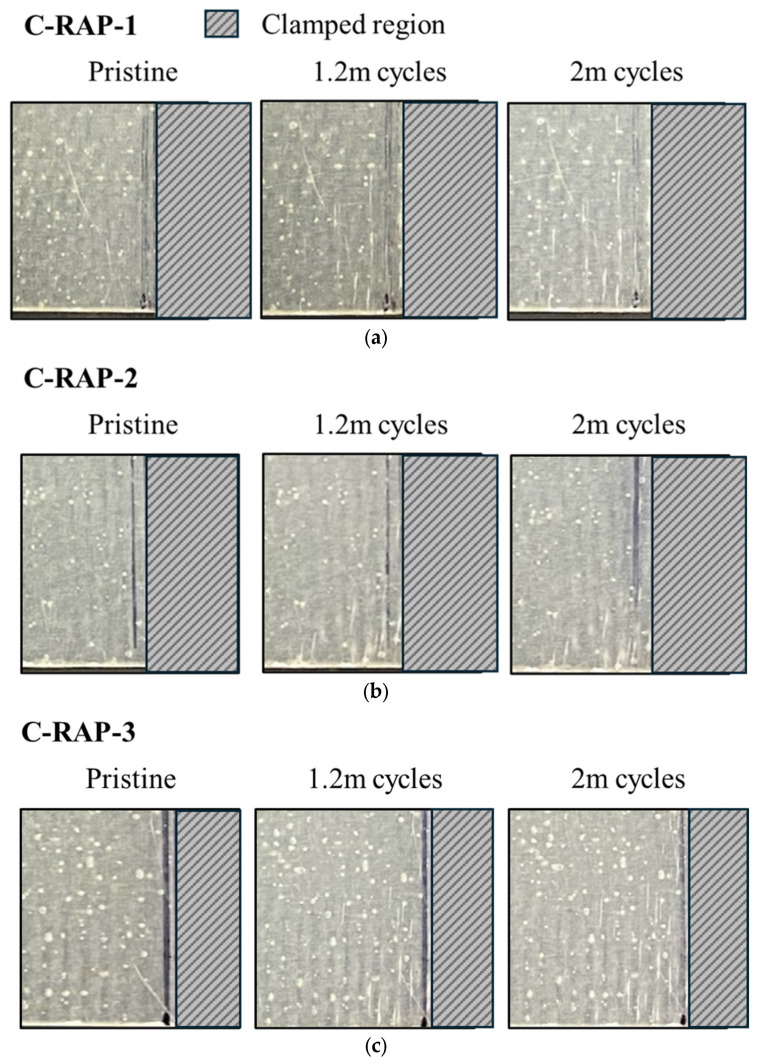
Damage accumulation and related fatigue cycles on (**a**) Specimen-C-RAP-1; (**b**) Specimen-C-RAP-2; (**c**) Specimen-C-RAP-3.

Upon closer examination of the substrate damage depicted in Figure 16, a whitened region is evident, gradually propagating from the clamped region on the right side towards the vicinity of the antenna patch. In Specimen-C-RAP-2, damage occurred primarily around the edges of the specimen, whereas Specimen-C-RAP-1 and Specimen-C-RAP-3 exhibited relatively larger whitened areas. The smaller extent of damage observed in Specimen-C-RAP-2 corresponded with a lesser decrease in resonant frequency, clearly indicating that the extent of damage correlates with the magnitude of frequency decrease.

#### 3.3.2. Configuration C: Surface-Mounted Antenna Patch (SMAP)

**Figure 17:**
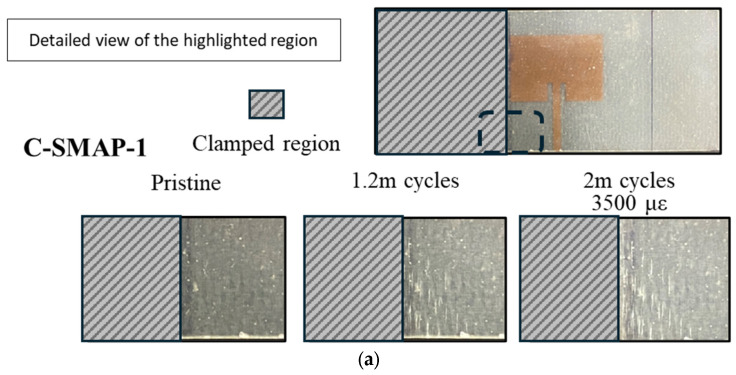

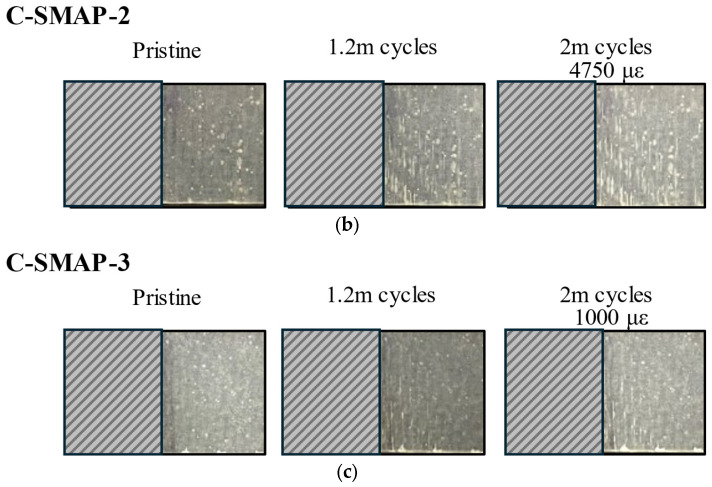
Damage accumulation and related fatigue cycles on (**a**) Specimen-C-SMAP-1; (**b**) Specimen-C-SMAP-2; (**c**) Specimen-C-SMAP-3.

**Figure 18:**
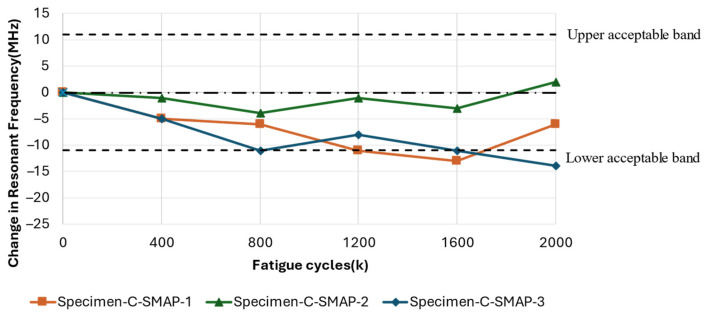
Resonant frequency shift of the CLAS due to fatigue of the substrate with Specimen Configuration C: SMAP.

Figure 17 shows the progression of damage on the substrate around the antenna. At 1.2 million cycles, Specimen C-SMAP-2 exhibited the most severe damage among the three specimens, while Specimen C-SMAP-3 showed a less noticeable whitened region, indicating limited damage. If the effect of cyclic fatigue on the substrate was the sole factor influencing shifts in resonant frequency, one would expect Specimen C-SMAP-2 to display the most significant frequency change at 1.2 million cycles and Specimen C-SMAP-3 the least. However, the results in Figure 18 demonstrate a different trend. At 1.2 million cycles, Specimen C-SMAP-1 exhibited the most significant decline in resonant frequency, reaching −11 MHz, while the resonant frequency of Specimen C-SMAP-2 showed limited change, remaining around the 0 MHz line. This trend contradicts findings from the literature and our C-RAP test results, where mechanical fatigue loading was applied only to the substrate, and substrate damage led to a decrease in resonant frequency.

**Figure 19:**
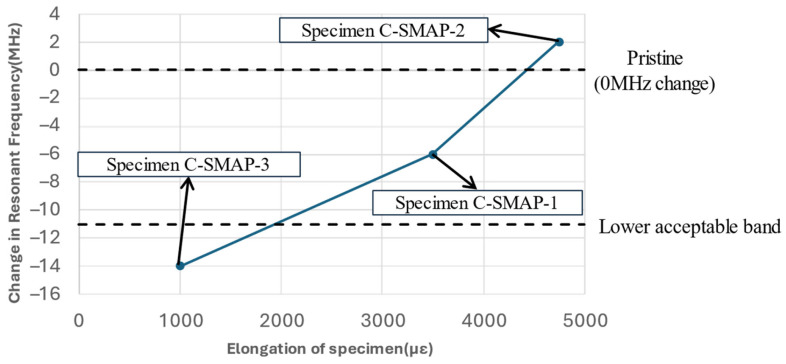
Resonant frequency shift of the CLAS with respect to the residual elongation of specimens.

The antenna patch in Configuration C: SMAP will experience fatigue loading along with the substrate due to their secure bonding. Figure 17 shows the residual elongation of the antenna patch after 2 million fatigue cycles. The residual elongation, reported in strain, for Specimens C-SMAP-1, C-SMAP-2, and C-SMAP-3 are 3500 με, 4750 με, and 1000 με, respectively. Figure 19 presents the resonant frequency of the CLASs with respect to their residual strain, which agrees with Equation (1) and the simulation work by Healey et al. [2], as the residual strain is equivalent to an increase in the dimension a of the patch antenna shown in Figure 2.

Therefore, during the cyclic fatigue tests with this configuration, the effect of mechanical elongation of the antenna, which increases the resonant frequency, might co-occur with substrate damage, which decreases the resonant frequency. This competition between the two effects leads to shifts in the resonant frequency that differ from the expected pattern based solely on substrate damage.

Furthermore, the results presented in Figure 17 show that the order of damage severity at 2 million cycles remained consistent with that observed at 1.2 million cycles, with Specimen C-SMAP-2 showing the most significant damage and Specimen C-SMAP-3 exhibiting the least. The elongation of the specimens further confirmed this order. However, Specimen C-SMAP-3 demonstrated a general decreasing trend with the most significant decrease of −14 MHz in resonant frequency, followed by C-SMAP-1 and then C-SMAP-2. The correlation between specimen elongation at 2 million cycles and the shift in resonant frequency deviates from the established monotonic correlations for frequency changes in the previous section and literature. This result suggests that during the initial fatigue cycles, the effect of substrate damage on resonant frequency is dominant, leading to a decrease. However, with increasing fatigue cycles, even though substrate damage continues to expand, the mechanical loading effect on the antenna becomes more pronounced, ultimately leading to an increase in the resonant frequency at 2 million cycles. The relationship between substrate damage severity, specimen elongation, and resonant frequency shifts for Configuration C: SMAP highlights the competing effects of geometry changes induced by mechanical loading on the antenna and the impact of substrate damage.

## 4. Discussion

According to Equation (1), the resonant frequency of the CLAS system is predominantly influenced by the antenna’s physical geometry and the substrate’s electromagnetic properties. Previous studies have demonstrated that mechanical loading significantly alters CLAS’s performance when either the antenna patch’s geometry or the substrate sustains damage. Specifically, the resonant frequency positively correlates with tensile strain applied along the antenna patch’s dimension, a [2]. Additionally, mechanical loading induces changes in the substrate’s relative permittivity ($\epsilon_{r}$) due to damage accumulation which, in turn, impacts the resonant frequency of CLAS [14,16]. These findings underscore the critical need to understand the effects of mechanical loading on the electromagnetic performance of CLASs.

The research presented in this paper provides a systematic investigation of a CLAS system utilising a 6-ply [0/90] GFRP substrate, focusing on the resonant frequency shifts observed under various mechanical loading conditions. This research encompasses two mechanical loading scenarios: quasi-static uniaxial tensile testing (QS), which examines how the CLAS responds to significant elongation of the specimen, and cyclic fatigue testing (C), which focuses on highly localised damage in the substrate that gradually propagates toward the antenna patch.

### 4.1. Quasi-Static Tension Test (QS)

Three specimens with Configuration A: IAP underwent an identical testing procedure in the quasi-static tests. The antenna patch and the substrate were both subjected to mechanical strain. This specimen configuration induced two competing factors affecting the resonant frequency of the CLAS under tensile loading: (a) the geometry-induced increase in frequency [2] and (b) the material-induced increase in permittivity leading to a frequency decrease [14,16]. The results indicate that the dominant effect under uniaxial loading along the substrate’s principal direction is the increase in permittivity, causing a decrease in resonant frequency. Additionally, the resilience of the CLAS was evident as the resonant frequency remained within the acceptable bandwidth before specimen failure. However, the elongation observed in the quasi-static test is unlikely to occur in aerospace structures with integrated antennas.

### 4.2. Cyclic Fatigue Tests (C)

Fatigue tests were conducted on Configuration B: RAP, where the antenna patch was removed before testing, leaving only the substrate to experience fatigue damage independently. The antenna is re-installed onto the test specimen for measurement when the specimen has completed a defined number of fatigue cycles. The results consistently demonstrated a decrease in resonant frequency with increasing fatigue cycles. This frequency shift was strongly correlated with the extent of damage sustained by the substrate, highlighting a clear relationship between substrate damage severity and frequency change magnitude. These findings align with the previous literature, which identified competing factors, including factor (b), as mentioned above.

The results from Configuration C: SMAP, where the antenna was bonded to the substrate, exhibited complex behaviour. Resonant frequency shifts did not consistently correlate with the severity of substrate damage due to competing effects. This interaction led to unexpected trends, such as the most significant frequency decrease being observed in C-SMAP-3 (−14 MHz), despite showing the least visible damage. Conversely, C-SMAP-2, which exhibited considerable whitening damage near the clamping region, demonstrated minimal frequency change, remaining close to the 0 MHz line. As fatigue cycles extended to 2 million, the interplay between substrate damage and antenna elongation became increasingly apparent. In C-SMAP-1, the resonant frequency initially decreased due to substrate damage but subsequently increased as the mechanical effects on the antenna patch began to dominate, ultimately recovering within the acceptable bandwidth.

The differing resonant responses between RAP and SMAP configurations highlight the competing effects of substrate damage and antenna patch elongation. Understanding this interplay is critical for accurately assessing the overall performance of the CLAS under localised fatigue induced by operational loads.

### 4.3. Comparing CLAS with [±45]_6_ GFRP

The dielectric properties of GFRP under mechanical loading varied with fibre orientations, as illustrated in Figure 5 and Figure 6. Compared with our previous work presented in [15], where we reported on a similar set of experiments with a 6-ply [±45] layout, we observed notable differences in the results of quasi-static tensile tests. However, the outcomes under cyclic fatigue loading were comparable between the GFRP specimens prepared with the two different ply configurations.

Under significant elongation from quasi-static tensile loading, the resonant frequency of the specimen prepared by [0/90] GFRP was reported to decrease with increasing residual strain. In contrast, for [±45] GFRP, the frequency initially decreased, then increased, and eventually shifted out of the operational bandwidth. These two distinct trends can be attributed to differences in how permittivity responds to mechanical loading at different fibre orientations of GFRP, highlighting a common underlying mechanism: under conditions of significant elongation, the substrate’s determining factor for resonant frequency shifts is the permittivity.

We attribute this difference in dielectric response to the extensive damage accumulated in the matrix material for [±45] GFRP. In contrast, for [0/90] GFRP substrate, the applied loading is taken predominantly by the fibre, and the specimen will not elongate as much as that in [15]. It is interesting to note that the limited matrix deformation reduced the resonant frequency of the CLAS. This observation underscores the influence of the substrate’s dielectric properties, which are directly impacted by damage accumulation. When the substrate alone was subjected to cyclic loading (Configuration B: RAP), both [0/90] and [±45] GFRP displayed a similar response: a general decrease in resonant frequency. This behaviour was attributed to an increase in permittivity, which corresponded to the initial damage accumulation on the substrate, aligning with the previous literature [10,14,16]. Furthermore, the results from specimens with Configuration C: SMAP for both types of GFRP revealed comparable fluctuations and a slight decreasing trend in frequency with increasing fatigue cycles. These consistent observations indicate that, during the initial stages of damage accumulation in the CLAS system, competing effects are present regardless of the substrate’s fibre configuration. The results presented in this paper and those in [15] show that the parameters in Equation (1) must be accounted for when designing CLASs. The material response to the accumulation of damage, whether static or fatigue, and its corresponding impact on its relative permittivity must be considered to achieve a damage-tolerant CLAS.

These findings establish a clear relationship between damage progression sustained by the CLAS system and the resonant frequency shifts in the CLAS. These results offer critical insights into the effects of quasi-static tension and cyclic fatigue loading on CLAS performance, underscoring the importance of a good understanding of the material behaviour and the effects of mechanical loading on the physical dimensions. The presented results set the foundation for interpreting future experimental data and enhance the understanding needed to predict these structures’ service life and reliability.

Building on these findings, future research should explore the competing effects of mechanical loading on the antenna patch and substrate and the potential impact of pre-existing defects and moisture exposure in the CLAS system. Such investigations could provide deeper insights into the interplay of these factors, further enhancing reliability predictions and supporting the development of robust designs for CLAS systems.

## 5. Conclusions

This study systematically investigated the effects of mechanical loading on the electromagnetic performance of the CLAS, with resonant frequency shifts serving as the primary performance indicator. The research examined two mechanical loading scenarios: quasi-static tensile testing and cyclic fatigue. The results demonstrate that substrate permittivity and mechanical deformation changes significantly impact CLAS’s resonant frequency.

In the quasi-static tests, the dominant influence of increased permittivity due to substrate damage resulted in a general decrease in resonant frequency. Variations in failure modes across specimens revealed the critical role of load distribution in determining performance. Cyclic fatigue tests further highlighted the interplay between substrate damage and antenna elongation. The RAP configuration isolates substrate effects and confirms a consistent correlation between the severity of substrate damage and frequency decrease. Conversely, the SMAP configuration exhibited complex behaviour, with competing effects of substrate damage and antenna elongation causing unexpected trends in resonant frequency shifts.

In addition, compared with [±45]_6_ GFRP, the CLAS with [0/90] GFRP predominantly bears loading through the fibres, resulting in less elongation. Differences in resonant behaviour underscore the need to fully account for all parameters in Equation (1), including material response to damage and its effect on relative permittivity, to design a damage-tolerant CLAS.

These findings establish a fundamental understanding of the relationship between damage progression sustained by the CLAS system and its performance under mechanical loading. The research underscores the importance of understanding the competing factors to ensure CLAS’s reliability and service life in aerostructures. This research provides a solid foundation for future experimental investigations and offers insights critical to advancing CLAS technology for practical applications.

## Data Availability

The datasets presented in this article are not readily available because the data are part of ongoing research. Requests to access the datasets should be directed to wing.kong.chiu@monash.edu.

## References

[1] Beziuk G., Krajewski A., Baum T.C., Nicholson K.J., Ghorbani K. (2023). Electromagnetic and Electronic Aerospace Conformal Load-Bearing Smart Skins: A Review. IEEE J. Microw..

[2] Healey R., Nicholson K.J., Wang J., Patniotis J., Lynch T. (2022). Conformal Load-Bearing Antenna Structures—Mechanical Loading Considerations. Sensors.

[3] Callus P.J. (2007). Conformal Load-Bearing Antenna Structure for Australian Defence Force Aircraft.

[4] Ahamed J., Joosten M., Callus P., John S., Wang C.H. (2016). Ply-interleaving technique for joining hybrid carbon/glass fibre composite materials. Compos. Part A Appl. Sci. Manuf..

[5] Li Z., Haigh A., Soutis C., Gibson A., Sloan R. (2017). Dielectric constant of a three-dimensional woven glass fibre composite: Analysis and measurement. Compos. Struct..

[6] You C.S., Hwang W. (2005). Design of load-bearing antenna structures by embedding technology of microstrip antenna in composite sandwich structure. Compos. Struct..

[7] You C., Hwang W., Park H., Lee R., Park W. (2003). Microstrip antenna for SAR application with composite sandwich construction: Surface-antenna-structure demonstration. J. Compos. Mater..

[8] Sharma S., Tripathi C., Rishi R. (2017). Impedance matching techniques for microstrip patch antenna. Indian J. Sci. Technol..

[9] Balanis C. (2016). Antenna Theory: Analysis and Design.

[10] Raihan R., Adkins J.-M., Baker J., Rabbi F., Reifsnider K. (2014). Relationship of dielectric property change to composite material state degradation. Compos. Sci. Technol..

[11] Raihan R., Reifsnider K., Cacuci D., Liu Q. (2015). Dielectric signatures and interpretive analysis for changes of state in composite materials. ZAMM-J. Appl. Math. Mech./Z. Für Angew. Math. Und Mech..

[12] Raihan R., Rabbi F., Vadlamudi V., Reifsnider K. (2015). Composite materials damage modeling based on dielectric properties. Mater. Sci. Appl..

[13] Vadlamudi V., Raihan R., Reifsnider K. (2017). Multiphysics based simulation of damage progression in composites. Mater. Sci. Appl..

[14] Vadlamudi V., Shaik R., Raihan R., Reifsnider K., Iarve E. (2019). Identification of current material state in composites using a dielectric state variable. Compos. Part A Appl. Sci. Manuf..

[15] Lu S., Nicholson K.J., Patniotis J., Wang J., Chiu W.K. (2024). The Effects of Mechanical Loading on Resonant Response of a Conformal Load-Bearing Antenna System. Sensors.

[16] Raihan M.R. (2014). Dielectric Properties of Composite Materials During Damage Accumulation and Fracture. Doctoral Dissertation.

[17] Lu S., Nicholson K., Patniotis J., Wang J., Kong W. (2023). Fatigue response of conformal load bearing antenna structures. Mater. Res. Proc..

[18] Kim H., Park M., Hsieh K. (2006). Fatigue fracture of embedded copper conductors in multifunctional composite structures. Compos. Sci. Technol..

[19] Kim H., Hsieh K. (2012). Measurement and prediction of embedded copper foil fatigue crack growth in multifunctional composite structure. Compos. Part A Appl. Sci. Manuf..

